# Jobs, Housing, and Mask Wearing: Cross-Sectional Study of Risk Factors for COVID-19

**DOI:** 10.2196/24320

**Published:** 2021-01-11

**Authors:** Eline M van den Broek-Altenburg, Adam J Atherly, Sean A Diehl, Kelsey M Gleason, Victoria C Hart, Charles D MacLean, Daniel A Barkhuff, Mark A Levine, Jan K Carney

**Affiliations:** 1 Department of Radiology Larner College of Medicine University of Vermont Burlington, VT United States; 2 Department of Medicine Larner College of Medicine University of Vermont Burlington, VT United States; 3 Department of Microbiology and Molecular Genetics Larner College of Medicine University of Vermont Burlington, VT United States; 4 Division of Emergency Medicine Larner College of Medicine University of Vermont Burlington, VT United States

**Keywords:** COVID-19, risk of infection, community exposure, self-protecting behavior, mask wearing, infection fatality rate, infection, self-protecting, mask, fatality rate, exposure, virus, SARS-CoV-2

## Abstract

**Background:**

Many studies have focused on the characteristics of symptomatic patients with COVID-19 and clinical risk factors. This study reports the prevalence of COVID-19 in an asymptomatic population of a hospital service area (HSA) and identifies factors that affect exposure to the virus.

**Objective:**

The aim of this study is to measure the prevalence of COVID-19 in an HSA, identify factors that may increase or decrease the risk of infection, and analyze factors that increase the number of daily contacts.

**Methods:**

This study surveyed 1694 patients between April 30 and May 13, 2020, about their work and living situations, income, behavior, sociodemographic characteristics, and prepandemic health characteristics. This data was linked to testing data for 454 of these patients, including polymerase chain reaction test results and two different serologic assays. Positivity rate was used to calculate approximate prevalence, hospitalization rate, and infection fatality rate (IFR). Survey data was used to analyze risk factors, including the number of contacts reported by study participants. The data was also used to identify factors increasing the number of daily contacts, such as mask wearing and living environment.

**Results:**

We found a positivity rate of 2.2%, a hospitalization rate of 1.2%, and an adjusted IFR of 0.55%. A higher number of daily contacts with adults and older adults increases the probability of becoming infected. Occupation, living in an apartment versus a house, and wearing a face mask outside work increased the number of daily contacts.

**Conclusions:**

Studying prevalence in an asymptomatic population revealed estimates of unreported COVID-19 cases. Occupational, living situation, and behavioral data about COVID-19–protective behaviors such as wearing a mask may aid in the identification of nonclinical factors affecting the number of daily contacts, which may increase SARS-CoV-2 exposure.

## Introduction

Since the global outbreak of SARS-CoV-2 (and the disease it causes, COVID-19), there has been significant research interest in understanding the disease’s ability to spread within populations. However, understanding the spread of COVID-19 has been particularly challenging because of asymptomatic spread [1-3]. With new rapidly developing assays capable of identifying serum antibodies, some regions and countries have launched investigations to identify the prevalence of asymptomatic infection. Studies that have collected seroprevalence data have used it to test the sensitivity and specificity of an enzyme immunoassay and microneutralization assay in Hong Kong [4], to combine and evaluate targeted testing and population screening in Iceland [5], and to compare incidence and infection fatality rates in the worst-hit towns in Germany after a superspreading event [6]. What these studies have in common is that they are aimed at improving epidemiological models of how the virus spreads and evaluating its transmission behavior. They include important indicators such as age, gender, and pre-existing conditions, as well as recent travel [5] and household size [6]. These studies help identify the proportion of the population at risk, increase understanding about hospitalization and fatality rates, and help guide decision making regarding strategies to control the pandemic. Other studies have focused on environmental and behavioral factors in the population without knowing infection rates in the same population [7-9].

Less studied is how environmental and behavioral factors such as occupation, housing situation, and COVID-19–protective behaviors affect infection rates. To date, most studies have focused on demographic risk factors among those who have tested positive for the virus [6,10]. Individual protective behaviors like wearing a mask are seldom studied in the COVID-19 literature [8]. There are two recent studies that reported important differences in mask-wearing practices between countries during COVID-19 pandemic, including countries in the East and West [11], and two neighboring countries (The Netherlands and Belgium) [12].

The objective of this study was to measure the prevalence and incidence of COVID-19 in the hospital service area (HSA) of the University of Vermont Medical Center (UVMMC), identify factors that may increase or decrease the risk of infection and exposure, and to analyze factors that increase the number of daily contacts. UVMMC is the largest hospital and most densely populated county in a rural state in the northeastern United States and its HSA is the area of the local community that is intended to be served by the hospital. We evaluated the prevalence of SARS-CoV-2 among community-dwelling adults in the most densely populated county in Vermont after the height of the COVID-19 pandemic in June 2020 and explored the environmental and behavioral factors associated with the risk of infection. At the time of this study, Vermont had a very low rate of COVID-19 infection. Active disease rates in the population were low and remained low throughout recent months. We conducted our study in Vermont, because we were able to obtain data from a representative sample of the most densely populated county in the state, accounting for approximately one-third of the total population of the state. We hope that this study serves as an example for more studies linking COVID-19 seroprevalence in the general population to behavioral data potentially affecting the spread of COVID-19.

This research combined individual survey data on COVID-19 risks and social behaviors with polymerase chain reaction (PCR) testing results from nasopharyngeal swabs and two different serologic assays. The addition of biological testing to known epidemiological data allowed for the calculation of accurate population prevalence rates, the true hospitalization and infection fatality rates, and inferences about exposure to the virus that may have more widespread implications.

## Methods

### Recruitment

Our sampling frame included community members from Chittenden county in the HSA of UVMMC who had an encounter with their primary care provider in the past 3 years. Using electronic health records, we randomly selected 12,000 individuals aged 18-70 years who had at least one primary care visit during the preceding 3 years, stratified by age and gender.

Individuals were contacted via email in two waves between April 30 and May 13, 2020, and asked to consent to participate in the survey.

After completing the survey, an offer was sent to these 1694 participants to receive PCR and serologic testing. To prevent recruitment bias among people who may have been motivated to obtain COVID-19 testing, participants were not aware of this optional testing component when filling out the survey.

### Survey

The survey instrument was developed by an international group of researchers and previously used to collect data from different countries [7]. The information collected included work and living situations, income, COVID-19–protective behaviors (such as wearing a face mask), beliefs about the COVID-19 pandemic and exposure to the virus, sociodemographic characteristics, and prepandemic health status. The survey also gathered specific information from respondents about the type of industry in which they are employed and their precise profession within that industry. Respondent profession was linked to profession exposure data derived from data from the US Department of Labor/Employment and Training Administration’s Occupational Information Network (O*NET) survey, which categorizes the level of exposure to disease/infections for a wide range of professions [13]. This O*NET measure has also been used by others linking job exposure to COVID-19 [7]. Scores range from 0 to 100, where 0 is “never,” 50 is “once a month or more but not every week,” and 100 is “every day.” Survey data was collected and stored via REDCap.

### COVID-19 Tests

COVID-19 prevalence (active infection) was tested with PCR testing on nasopharyngeal swabs, while incidence rate was tested using two different serologic assays performed on patient-matched blood samples. The PCR test detects the genetic code for the SARS-CoV-2 virus (which causes COVID-19) and identifies active COVID-19 infection. The serologic tests detect antibodies to COVID-19 and indicate whether the participant has mounted an immune response to the virus. COVID-19 prevalence (active infection) was tested at the State of Vermont Department of Health Laboratory by PCR using the TaqPathTM COVID-19 Combo Kit (ThermoFisher, catalog numbers A47813 and A47814) on ribonucleic acid (RNA) extracted from nasopharyngeal swabs. This assay was granted Emergency Use Authorization [14], and uses primer sets targeting the ORF1ab, nucleocapsid, and spike regions of the SARS-CoV-2 genome. Each assay includes a positive SARS-CoV-2 RNA control (50 copies per reaction), a negative (diluent-only) control, and an MS2 phage as an internal process control for nucleic acid extraction. Briefly, RNA was extracted from nasopharyngeal swabs, reverse transcribed using the one-step multiplex Mastermix, and assessed on an Applied Biosystems 7500-Fast Dx PCR instrument as listed in the product manual using a sample cycle threshold (CT) cutoff of ≤37 for the calling of positives.

Serologic testing was done on separated serum (BD SST catalog number 367977) using two different assays granted Emergency Use Authorization by the Food and Drug Administration: (1) the VITROS (Ortho Clinical Diagnostics) anti–SARS-CoV-2 IgG test conducted by the Mayo Clinic and (2) an open-source laboratory-developed two-step enzyme-linked immunosorbent assay (ELISA) originally developed by the Mount Sinai School of Medicine [15] and conducted at the University of Vermont Larner College of Medicine. Both assays exhibit ≥90% sensitivity and 100% specificity with ≥99.5% negative predictive value (NPV) at a prevalence of 5% [16]. The two-step IgG ELISA was recently validated to over 99% sensitivity in samples from patients with COVID-19 [17]. Serology for the receptor binding domain of the SARS-CoV-2 spike protein (RBD-S) has been shown to exhibit extremely low cross-reactivity for other non-SARS coronaviruses [18] and to correlate with neutralization activity [17,19], making it a highly specific and relevant measure of SARS-CoV-2 infection.

### Statistical Analysis

The testing results were merged with the survey data. Observations that had missing values for key variables were deleted (n=19), which left us with a total sample size of 435 for the multivariate analysis. We had two outcome variables in the analysis. The first was whether or not the person tested positive for COVID-19 antibodies. The second was the number of contacts the person had on a “typical” day (<18, 18-64, and >64) during the two weeks prior to the survey.

For the dichotomous outcome variable (whether a participant had a positive COVID-19 antibody test), we performed multivariate analyses using Probit models. The count data representing the number of daily contacts for the participants followed a Poisson distribution: the number of people seen outside the household can be seen as rare events, since many respondents did not see others at all. As the Poisson distribution assumes that the mean and variance are the same, we tested the fit of a Poisson model versus negative binomial models [15]. The likelihood ratio test is a test of the overdispersion parameter α: when α is zero, the more flexible negative binomial distribution is equivalent to a Poisson distribution. In our case, α was significantly different from zero, suggesting the negative binomial distribution was appropriate, so we used nbreg in Stata 16.0 (StataCorp) to analyze the number of daily contacts. We used a Vuong test of the zero-inflated model versus the standard model [16,20] and found that the excess zeros should not be modeled independently. We ran different models for number of contacts with children, adults, and older adults. We used robust standard errors for the negative binomial models. Statistical analysis was performed in Stata, including descriptive statistics and multivariate analysis.

Key control variables included age (because of the relatively small sample size of positives, age was dichotomized to over and under 45 years), income (in categories, and dichotomous >$100,000/<$100,000), gender, education (college yes/no), and presence of chronic illnesses (yes/no from a list including conditions identified by the Centers for Disease Control and Prevention as increasing the risk of COVID-19 complications, which included diabetes, high blood pressure/hypertension, heart disease, asthma or other chronic respiratory issues, allergies, and kidney disease or other chronic illnesses that require long-term care from a doctor). We also included variables indicating whether the participant had lost their job due to COVID-19 and whether their work situation had changed (working from home instead of previous location), whether they had been tested before, what symptoms they had and whether they sought testing for those symptoms, whether they had been diagnosed, whether they had pre-existing conditions, and whether they had been in contact with others who had tested positive.

### Human Subjects Research Review Statement

This study has been approved by the Institutional Review Board of the University of Vermont. We received separate approval for the survey study and the COVID-19 testing study.

Study participants signed eConsent forms for both the survey part and the testing part of the study. There was no compensation for participation in this study.

The health information of participants is protected by a federal law called the Health Information Portability and Accountability Act (HIPAA). The study team stored the data from the survey and COVID-19 tests in a safe environment. Only the research team, the UVM Institutional Review Board, and state and federal agencies that oversee research have access to this information. No identifying data was made available to any other sources.

## Results

### Participation

A total of 12,000 patients were invited to participate in this study. All individuals were provided with an opportunity to opt out of the survey at any time during the study. A total of three follow-up reminders were sent. Of this initial sample of 12,000 individuals, 98% had functioning email addresses (n=11,700); the response rate was 19.4% (n=2275), and 75% of these respondents both read the consent form and agreed to participate (n=1961 participants). Of these, 86.4% completed the survey, for a total of 1694 respondents (14.4% of the initial sample). All 1694 survey respondents were invited to opt in to COVID-19 testing. A total of 26.8% (n=454) of participants provided samples between June 25 and June 28, 2020.

### COVID-19 Test Results

In total, 10 of 454 participants tested positive for IgG antibodies in a two-step serologic assay in which samples with presumed IgG reactivity against the RBD-S are confirmed in an independent assay wherein the IgG endpoint titer against the full-length SARS-CoV-2 spike protein is determined. Of the 10 samples, 6 were confirmed by the VITROS SARS-CoV-2 IgG assay, which detects an undisclosed antigen from SARS-CoV-2 and provides a nonquantitative positive/negative result. These 6 samples exhibited an average anti–RBD-S optical density (OD) of 0.91 (SD 0.22; range 0.64-1.28) and anti-spike reciprocal IgG titers of 21,300 (SD 27,500; range 900-72,900) in the two-step assay performed at the University of Vermont. The remaining 4 exhibited an OD of 0.56 (SD 0.55; range 0.18-1.38) and titer of 350 (SD 380; range 100-900). There was not a statistically significant difference (*P*=0.2 for OD and *P*=0.17 for titer by paired Student *t* test) between the 6 UVM/VITROS-positive and 4 UVM-only positive samples. Furthermore, all positive samples by two-step IgG assay met the assay positivity cutoff requirements (Step 1: RBD-S OD ≥2-fold over background, which was ~0.08 and Step 2: titer≥80). The positivity rate for antibodies against SARS-CoV-2 in our catchment area was therefore 2.2% (95% CI 0.8%-3.6%). Only 1 participant (0.2%) tested positive for active SARS-CoV-2 replication using the nasopharyngeal swab.

Extrapolating these serology results to the 164,572 residents of the county, approximately 3621 have been infected by COVID-19 so far (95% CI 1317-5925). The State Department of Health reported a total of 662 positive cases in the same county at the time the study test samples were obtained. This implies that 18.3% of positive cases have been identified by the existing community-based testing (95% CI 11.2%-50.3%).

From the onset of the COVID-19 pandemic to the time of our data collection, 50 individuals from the county had been hospitalized at UVMMC. This implies that 1.4% of persons with COVID-19 required hospital care during the March-July 2020 time frame (95% CI 0.8%-3.8%). At the time of study completion, there have been a total of 39 deaths attributed to COVID-19 in Vermont, which implies an infection fatality rate of 1.1% (95% CI 0.7%-3.0%). Of the 39 deaths, 19 (48.7%) were in nursing homes. If these deaths are excluded, we calculate a case fatality rate of 0.55%.

We did not perform statistical analyses with the PCR results, because we only found 1 positive PCR test and therefore did not have enough statistical power for analysis.

### Factors Associated With Positive SARS-CoV-2 Test

Table 1 shows the association between positive serology for SARS-CoV-2 and select sociodemographic factors. The number of contacts with both adults and older adults was statistically significantly higher for those who tested positive than those who did not (5.0 versus 31.6, *P*<.001 and 2.9 versus 14.8, *P*<.001, respectively). There was no statistically significant relationship for the number of contacts with children. Similarly, the number of contacts with people who tested positive was higher for the COVID-19 population (0.9) versus the negative subjects (0.1; *P*<.001). There were no statistically significant differences between those who tested positive and those who did not in average age, gender, number of reported symptoms, work exposure, urbanity, living environment, or mask wearing outside work.

**Table 1:** Descriptive statistics from the sample of 435 survey respondents showing frequencies and percentages in different categories of risk factors associated with COVID-19 infection in Vermont between April 20 and May 13, 2020.

Respondent characteristic	COVID-19–negative subjects (n=425), mean or proportion (SD)^a^	COVID-19–positive subjects (n=10), mean or proportion (SD)^a^	*T* statistic	*P* value
Number of contacts with children	1.5 (0.3635)	1.0 (0.4629)	0.1754	.86
Number of contacts with adults	5.0 (0.6383)	31.6 (20.1112)	–5.0571	<.001
Number of contacts with older adults	2.9 (0.6565)	14.8 (7.4276)	–2.4882	.01
Number of contacts with people who tested positive for COVID-19	0.1 (0.4247)	0.9 (1.3562)	–1.8110	.07
Age (years)	51.4 (0.6366)	51.9 (3.6028)	–0.1075	.91
Sex (female=1)	0.6 (0.0241)	0.6 (0.1830)	–0.2230	.82
High income (1=yes)	0.6 (0.0243)	0.4 (0.1830)	0.9561	.34
Any symptoms (1=yes)	0.2 (0.0180)	0.5 (0.1890)	–2.6804	.007
Diabetes (1=yes)	0.04 (0.0100)	0.13 (0.1250)	–1.1380	.26
Exposure at work, Occupational Information Network score (1-100)	26.2 (1.8328)	19.0 (12.0208)	0.5159	.61
Urban (versus suburban/rural; 1=yes)	0.3 (0.0218)	0.3 (0.1637)	0.1638	.87
Live in condominium/apartment (versus house; 1=yes)	0.2 (0.01923)	0.3 (0.1637)	–0.4041	.69
Wearing mask outside work (1=yes)	0.7 (0.230)	0.5 (0.1890)	1.1382	.26

^a^Based on a *t* test, Ha: diff!=0.

### Regression Results

Table 2 presents the results of the Probit regressions examining factors associated with positive COVID-19 test results. The three columns represent the different models. The first shows the effect of the number of daily contacts with children, the second shows the effect of the daily number of contacts with adults, and the third shows the effect of the daily number of contacts with older adults (>65 years). We used generally accepted standards for children (those aged <18 years), older adults (those aged ≥65 years), and adults (those aged 18-64 years). We found that with every additional adult that participants would see on a daily basis, they had a 1.2% (*P*<.05) higher probability of getting a positive test result. For contact with older adults, this increased probability was the same (1.2%, *P*<.05). With each additional contact with a person who had tested positive for COVID-19, participants had a 44.1%-53.6% (*P*<.05) higher probability of testing positive for the virus. Those aged >45 years had a 20.4%-24.8% higher probability of infection with each additional contact. We found no other covariates to be statistically significant in our models.

**Table 2:** Predicted probabilities of COVID-19 infection in Vermont between April 20 and May 13, 2020.

Respondent characteristic	Model 1: probability of COVID-19 infection: children contact model^a^	*P* value	Model 2: probability of COVID-19 infection: adult contact model^a^	*P* value	Model 3: probability of COVID-19 infection: older adult contact model^a^	*P* value
Number of contacts with children (those aged 0-17 years)	–0.0104 (0.0403)	.88	N/A^b^	N/A	N/A	N/A
Number of contacts with Adults (those aged 18-64 years)	N/A	N/A	0.0118 (0.0059)	.03	N/A	N/A
Number of contacts with older adults (those aged ≥65 years)	N/A	N/A	N/A	N/A	0.0122 (0.0063)	.05
Number of contacts with people who tested positive for COVID-19	0.5359 (0.2111)	.04	0.4406 (0.2243)	.16	0.5356 (0.2123)	.42
Aged ≥45 years	0.2038 (0.3701)	.36	0.2432 (0.3854)	.27	0.2484 (0.3846)	.29
Female	–0.0766 (0.3404)	.77	0.0501 (0.3632)	.53	–0.0025 (0.3567)	.65
High income	–0.3967 (0.3402)	.15	–0.3377 (0.3480)	.18	–0.4023 (0.3484)	.14
Any symptoms	0.3969 (0.3579)	.08	0.4159 (0.3703)	.07	0.4052 (0.3661)	.07
Diabetes	0.1394 (0.6342)	.96	–0.4759 (0.9156)	.46	–0.1672 (0.7184)	.68
Observations	413	N/A	413	N/A	413	N/A

^a^Probit regression marginal effects with standard errors in parentheses. Pseudo *R*^2^: 0.42.

^b^N/A: not applicable.

Table 3 presents the results of the negative binomial models reporting factors affecting the number of daily contacts. As expected, the more work exposure (as identified in the O*NET index), the more daily contacts participants would have. This is especially true for professions in which one sees more older adults. We also found that females saw almost one fewer adult per day than men did (β=0.88, *P*<.01) and that those living in an apartment or condominium rather than a house would see almost one adult more on a daily basis (β=0.78, *P*<.05). Interestingly, results showed that workers who wear masks outside of work also saw more adults than those who did not wear a mask outside of work (β=0.77, *P*<.01).

**Table 3:** Relationship between survey respondent characteristics and number of daily contacts in Vermont between April 20 and May 13, 2020.

Characteristics	Model 1: number of contacts with children^a^	*P* value	Model 2: number of contacts with adults^a^	*P* value	Model 3: number of contacts with older adults^a^	*P* value
Exposure at work	0.0214 (0.0123)	.08	0.0194 (0.0048)	<.001	0.0391 (0.0111)	.009
Female	–0.3636 (0.8061)	.70	–0.8757 (0.3395)	.004	0.1514 (0.8568)	.78
Aged ≥45 years	–0.1720 (0.7237)	.97	0.2602 (0.3877)	.63	1.3367 (0.9235)	.57
Urban (versus suburban)	0.7666 (0.9628)	.42	0.5204 (0.3646)	.16	1.0165 (1.2149)	.54
Living in condominium/apartment (versus house)	–1.6075 (0.9300)	.08	0.7765 (0.4091)	.08	0.4172 (0.9216)	.99
Wearing mask outside work	1.0897 (0.7637)	.10	0.7658 (0.2822)	.01	0.3159 (0.8215)	.85
Observations	49	N/A^b^	49	N/A	49	N/A

^a^Negative binomial estimates, standard errors in parentheses.

^b^N/A: not applicable.

## Discussion

### Principal Results

In this study, we evaluated the prevalence of SARS-CoV-2 among community-dwelling adults in the most densely populated county in Vermont after the height of the COVID-19 pandemic in June 2020, and explored the environmental and behavioral factors associated with the risk of infection. We found a seroprevalence rate of 2.2% and an infection fatality rate of 0.55% after excluding deaths in nursing homes. In the multivariate analysis, we found that the number of daily contacts with adults and older adults increased the probability of infection. Type of occupation, living in an apartment or condominium versus a house, and wearing a face mask outside work increased the number of daily contacts.

The main objective of this study was to identify the prevalence of COVID-19 in an asymptomatic (general) population and identify behavioral and environmental differences between the infected and the uninfected. There are some COVID-19 seroprevalence studies to date, such as one in Iceland [5], which included volunteers from the total population, and a nationwide study in Spain [21]. There are also a few studies among subpopulations, such as one among health care workers in Northern Italy [22], and regional populations in Hong Kong and China [4,23], the United States [10,24,25], and Switzerland [26]. Most of these studies selected participants randomly, but environmental factors potentially affecting the seroprevalence numbers were undetermined. Important predictors in COVID-19 predictive simulation models, such as to what extent social distancing had been practiced, were unknown in these studies. Therefore, a second goal of this study was to get a better idea of actual social distancing practices in our research area and use this data to better inform modelling efforts to predict infection and hospitalization rates. The uniqueness of this study is that it combines survey data with COVID-19 testing data, which has not been done in many other places. To our knowledge, there has been one other study linking seroprevalence data to survey data [27]; however, that study in Germany primarily focused on symptoms and did not include factors such as daily routines and behaviors. Although we acknowledge the limitations of our research study, we believe it serves as an example of how to effectively link behavioral and clinical data.

We were able to identify environmental and behavioral factors affecting the risk of contracting COVID-19. We found that seeing more children per day does not increase the probability of getting COVID-19, but having more daily contact with adults and older adults does. We further identified factors that have an increasing effect on the number of daily contacts, such as living in an apartment and wearing a mask.

### Limitations

Our study does have a number of limitations. One is the assumption that the prevalence rates from our sample are representative of prevalence rates for the Chittenden county population. Our sample may be nonrepresentative because of both the inclusion criteria (those with the University of Vermont Medical Center as their primary care destination) and exclusion criteria (those aged <18 years and >70 years and pregnant people). However, because we do not anticipate that the inclusion and exclusion criteria are correlated with disease prevalence in the community, our results are likely representative of the population.

There are a number of possible mechanisms that could create differences between our sample and the population and thereby potentially create bias in the estimates of community prevalence. There may be bias based on observable characteristics, as we drew our initial sample from a population that either has registered with a primary care physician or had a health event in the past 3 years. To test this possibility, we applied sample weights using Census population data for Chittenden county and found our results to be robust to weighted regression results. However, this does not address unobserved characteristics such as wealth and travel time, which may introduce selection bias. For example, if persons who believed they were infected were more likely to participate, this would create an upward bias in our estimates. To test this possibility, we estimated a weighted regression, including all survey respondents including those who received the test invitation but declined. We found no significant difference in the estimates using both populations (tested and not tested). It is also possible the results are biased based on unobserved differences, both between the sample and the population and between the survey sample and the prevalence sample. In the absence of an appropriate instrument, we could not test this effect. Specimen collection was done a little over one month after survey responses were completed. This lag in data collection may potentially pose a temporality issue in the analyses, especially related to risk factors and PCR positivity. However, our infection analysis did not focus on PCR positivity but on a positive serologic test, which addresses infection over a larger period of time.

Compared to other US states, the hospitalization rates for COVID-19 we calculated have been at the lower end of the predicted range of COVID-19 inpatient predictive models [17]. The IFR is at the higher end of reported population rates, largely driven by a high number of nursing home deaths. The data we collected finds approximately 1 out of every 100 individuals infected with COVID-19 in the county needed inpatient care. This provides a benchmark to use to anticipate future shortages of hospital capacity.

The state of Vermont has had a very low rate of COVID-19 infection since the beginning of the pandemic. Active disease rates in the population are currently very low and have been low throughout the duration of the pandemic. Although we were able to test a large sample for COVID-19, the number of positive cases was small, which limited the multivariate analysis. To simplify models, we dichotomized some covariates, thereby losing some more detailed information about the exact effect size of individual levels of the covariates.

By testing in the general population, estimations about the total number of infections in similar demographic areas with different infection rates can be made based on the IFR and the number of deaths. This facilitates the kinds of (inter)national comparisons that could be helpful for developing effective mitigation strategies. Comparing the IFR with numbers of officially reported infections can allow for more refined estimates of unreported cases, which is another data point that is important for understanding pandemic dynamics.

### Conclusions

This study has several important policy implications for contemplating different COVID-19 mitigation strategies. We found that the key factors associated with a higher probability of testing positive for COVID-19 were the number of contacts with adults and older adults, particularly contacts with people who have COVID-19. The factors that predict contacts, in turn, are working environment, living environment, and regularly wearing a mask outside of work. This study reinforces the concerns about risks for persons who have high levels of public contact during the pandemic. The finding of the increased risk associated with living in apartments/condominiums likely partially explains higher infection rates in large metropolitan areas (eg, New York City) and lower income communities.

The findings with regard to mask wearing are more concerning. With many states and governments now debating whether the use of face masks should become mandatory, more research is needed about the behavioral effects of mask wearing and other policy measures. A recent study showed that mask wearing is associated with a lower prevalence of depression, which may be explained by seeing more people [28]. Another study addressed specific measures in the work environment to prevent COVID-19 [29]. It is plausible that mandating masks could be counterproductive if the increased risk associated with an increase in contacts is larger than the decrease in risk associated with mask wearing. That is, it is possible masks may provide a false sense of security that leads to people letting their guard down and trusting the mask more than is warranted. Further research into the effectiveness of masks and behavioral responses to mask mandates is urgently needed.

## Abbreviations

IFR: infection fatality rate
HSA: hospital service area
NPV: negative predictive value
O*NET: US Department of Labor/Employment and Training Administration’s Occupational Information Network
OD: optical density
PCR: polymerase chain reaction
RBD-S: receptor binding domain of the SARS-CoV-2 spike protein
RNA: ribonucleic acid
UVMMC: University of Vermont Medical Center
